# Attentional Orientation Patterns toward Emotional Faces and Temperamental Correlates of Preschool Oppositional Defiant Problems: The Moderating Role of Callous-Unemotional Traits and Anxiety Symptoms

**DOI:** 10.3389/fpsyg.2017.01928

**Published:** 2017-11-07

**Authors:** Georgiana Susa Erdogan, Oana Benga, Crina Marină

**Affiliations:** Developmental Psychology Lab, Department of Psychology, Babes-Bolyai University, Cluj-Napoca, Romania

**Keywords:** attentional orienting to emotional faces, preschool children, temperament, oppositional defiant problems, callous unemotional traits, anxiety

## Abstract

The present study examined the independent contributions and the interaction effects of oppositional defiant problems (ODD), callous unemotional traits (CU) and anxiety symptoms on attentional orienting to emotional faces, in a community sample of preschoolers. Additionally, based on Rothbart's (2007) model of temperament, we analyzed whether fine-grained dimensions of reactivity (fear, anger, discomfort, sadness, activity level, approach, high intensity pleasure, impulsivity) and self-regulation (attentional shifting, attentional focusing, inhibitory control), as well as the higher order temperamental factors of negative affectivity, surgency and effortful control are associated with CU traits and ODD-related problems. Attentional orienting to emotional faces was assessed with pictorial Dot-probe task, while teachers rated CU traits and ODD-related problems. Also, parents reported on ODD-related problems, anxiety and temperament. Results indicated significant interaction effects between ODD-related problems and CU, as well as between CU and anxiety, in predicting attentional orientation patterns for angry, fearful and happy faces. Moreover, temperamental reactivity was positively associated with CU traits and ODD-related problems, whereas temperamental self-regulation was negatively related to CU traits and ODD-related problems. Results of this study have implications for early intervention and prevention approaches targeting preschool oppositional defiant problems.

## Introduction

Children exhibiting elevated levels of disruptive behaviors [oppositional defiant disorder (ODD) and conduct disorder (CD)] and/or the problems from the broadband externalizing spectrum often follow a life-course trajectory of conduct problems (i.e., repetitive and persistent patterns of behavior that violate the rights of the others and major age-appropriate societal norms or rules, respectively) that place them at greater risk of later antisocial behavior during adolescence (Odgers et al., 2008; Hyde et al., 2013). Growing evidence suggests that disruptive behaviors have their roots in early childhood (as early as age 3) (Shaw and Gross, 2008; Hyde et al., 2013; Waller and Hyde, 2017), therefore more research focused on identifying early correlates of these problems is needed. Moreover, recent work has suggested that individuals with disruptive behaviors are a heterogeneous group (Frick and Nigg, 2012), and this may undermine effective prevention, intervention and treatment programs. For example, current research proposes that ODD (defined by symptoms of irritability and defiance of adult authorities) should be studied separately from CD (defined by specific antisocial behaviors, such as fighting, bullying, stealing, vandalism, and lying for personal gain), as the two disorders have different developmental trajectories and are associated with different risks (Lahey and Waldman, 2012). Consequently, studies that examine subgroups of children with disruptive disorders of different underlying etiologies, as early in development as possible, have the potential to inform more effective, personalized treatments. One recent approach to parsing disruptive behaviors into etiologically distinct subtypes is to measure the presence of callous-unemotional (CU) traits. CU traits include characteristics such as lack of remorse and guilt, shallow and deficient emotions, as well as an over-focus on reward and insensitivity to punishment, lack of empathy, which are all considered to be the core features of psychopathy (Frick et al., 2003; Frick and White, 2008). In children, measures of CU assess empathy and guilt deficits, as well as reduced emotional responsiveness to the feelings of others or threat cues. In DSM-5 (American Psychiatric Association, 2013), the inclusion of the specification “with limited prosocial emotions” for CD disorder allows the identification of a more homogenous subgroup of children with CD who also have CU traits. As for ODD, studies have shown, as early as preschool age, that, compared to children with low levels of CU traits and ODD, those with higher levels of CU traits have more severe ODD problems, showing deficits in processing emotional stimuli, such as fearful faces, having lower levels of fearfulness and anxiety, manifesting insensitivity to punishment and displaying physiological hypoarousal, such as low stress reaction—lower heart rate at rest and during reactivity to emotional stimuli (Fanti, 2016). Hence, there is general consensus that: (a) CU traits can be present before the CD disorder develops; (b) although these traits are distinct from ODD, they tend to co-occur across development (Frick et al., 2014) and are distinguishable in the first 3 years of life (Willoughby et al., 2011, 2014; Waller et al., 2015a,b). Further heterogeneity has been suggested based on the combination between anxiety and CU traits. More specifically, while the presence of CU traits without anxiety is characterized by difficulties in emotional responding to others' distress and by low stress reaction, the combination of high levels of CU and anxiety is characterized by negative emotionality, impulsivity, hyperarousal, high startle reactivity to emotional stimuli (Dackis et al., 2015) and high fear reactivity, aroused by environmental adversity (traumatic experience, lower income, abuse). In consequence, the current study aims to investigate, within a community sample of preschoolers, the cognitive correlates (attentional orientation patterns toward emotional faces) of combined ODD problems and CU traits, by also taking in consideration the role of anxiety symptoms. In addition, we were interested in analyzing temperamental correlates of both ODD problems and CU traits during this developmental period. Given that our aim was to disentangle (cognitive and temperamental) correlates of normative variation in disruptive behaviors, not confounded by the severity of conduct disorder, we chose to focus on ODD-related problems. ODD-related problems circumscribe less severe forms of defiant, disobedient, and uncooperative behaviors and age-inappropriate anger and irritability, respectively. Given the prevalence of all these behaviors starting from preschool age, we considered that an approach focused on them will be developmentally appropriate (Ezpeleta et al., 2017a).

### Attentional orienting to emotional faces in children as a function of ODD-related problems, CU traits and anxiety symptoms

There is a growing body of research into emotion recognition in different subgroups of children with disruptive behaviors. Collectively, these studies show that youth with conduct problems, particularly those with CU traits, have been reported to manifest impairment in expression recognition of fearful and sad faces, while the recognition of angry faces remains intact (see for a recent review Blair et al., 2016). Although children with higher CU traits and disruptive problems show a reduced recognition of fearful and sad faces, it is important to mention that increasing the intensity of an emotional stimulus—through morphing or by orienting the participant's attention toward the eyes—reduces or removes group differences in fearful and sadness recognition (Blair, 2013). However, very little research has focused on investigating attentional orientation toward emotional faces in these children. As recently suggested by Hodsoll et al. (2014), it is important to consider whether disruptive problems also involve changes in attention to emotional information. In real life situations, we tend to process emotional information alongside other stimuli, therefore it is critical for successful social functioning to react to emotional cues, even if these occur while we are engaged in another activity. In spite of its relevance for early ages, most research in the area of disruptive problems and attention to emotional information has focused on samples from late childhood and adolescence. For example, Hodsoll et al. (2014) used an attentional capture task, in which boys aged 8 to 16 with clinical levels of conduct problems and high levels of CU were asked to judge the orientation of a single male face that was displayed simultaneously with two female faces. In this task, three types of trials were presented: trials with only neutral faces, trials with an emotional distractor face and trials with an emotional target face. Trials with emotional distractor faces or emotional target faces presented images showing fearful, angry or happy expressions. Results showed that, as compared to typically developing children and children with low levels of CU traits, children with conduct problems and high levels of CU traits displayed reduced attentional capture by irrelevant emotional faces. Moreover, a study by Kimonis et al. (2006) used the Dot-probe paradigm (an attentional task that indexes attentional orientation patterns for emotional stimuli) with serious male adolescent offenders, revealing that those who had high levels of both CU traits and anxiety symptoms oriented significantly more their attention toward emotionally distressing pictures, as compared to those with high levels of CU traits but low anxiety, who were not engaged by these stimuli (Kimonis et al., 2012). Regarding early childhood data, the study conducted by Wagner et al. (2016) focused on the prediction that children with higher CU traits and ODD symptoms manifested during development have deficits in processing emotional relevant cues, such as gazing toward caregivers, as early as infancy. In their longitudinal study, these authors investigated infants' mother-directed gaze and reactivity during the face-to-face as well as still-face episodes of the still-face paradigm, performed at 6 months. This study revealed that infants' mother-directed gaze during the face-to-face episode predicted fewer ODD behaviors in early childhood. Additionally, their analyses suggested that infants' negative reactivity during the still-face episode predicted fewer ODD behaviors in early childhood. Also, mother-directed gaze during the face-to-face episode moderated the relation between negative reactivity during the still-face episode, early childhood ODD and CU behaviors, respectively. Specifically, mother-directed gaze attenuated the negative relation between reactivity, ODD, and CU behaviors. Moreover, studies conducted by Kimonis et al. (see Kimonis et al., 2006, 2012, 2016), although using stimuli from International Affective Picture System instead of emotional faces, within the Dot-probe task, are relevant in this respect. For example, Kimonis et al. (2016) showed that preschool children rated with high levels of CU traits and behavior problems oriented their attention less to distress cues (e.g., a crying child).

Taken together, the few available behavioral studies suggest that children with disruptive problems (ODD or CD) and higher levels of CU traits differ from children with disruptive problems but lower levels of CU, by showing less attentional orienting (i.e., engagement) to emotional faces. In contrast, children with both CU traits and anxiety symptoms tend to orient significantly more their attention toward emotionally distressing facial expressions.

Furthermore, neuroimaging studies investigating the neural correlates that underlie emotional processing deficits characteristic for youth with ODD problems, such as poor fear conditioning and impaired processing of emotional faces (Glenn and Raine, 2008; Hyde et al., 2013; Blair et al., 2014; Baker et al., 2015), have suggested divergent results. Majority of such studies have focused on amygdala reactivity, considered to be involved in these emotional deficits, and on clinical or forensic adolescent samples, dichotomously categorized on both conduct problems and CU traits. Several investigations found that conduct problems coupled with low levels of CU traits are associated with increased amygdala reactivity to fearful and angry facial expressions (Viding et al., 2012; Hyde et al., 2013; Blair et al., 2014; Sebastian et al., 2014), while those coupled with high levels of CU traits are correlated with decreased amygdala reactivity to emotional stimuli, particularly fearful facial expressions (Odgers et al., 2008; Jones et al., 2009). However, in a recent investigation with adolescents, Dotterer et al. (2017) investigated the links between amygdala reactivity to fearful and angry facial expressions, subclinical levels of antisocial behavior and CU traits. Their results showed that antisocial behavior was related to increased amygdala reactivity to angry facial expressions, whereas CU traits or the interaction between CU traits and antisocial behavior were not significantly related to amygdala reactivity for neither angry nor fearful faces. In contrast, Hyde et al. (2016a,b) found, this time in a sample of young, low-income, urban men, that antisocial behavior (but not CU traits) was negatively related to amygdala reactivity to fearful faces. In conclusion, neuroimaging studies that have examined the neural correlates of face processing in disruptive behavior problems have exclusively focused on conduct disorder and they did not take into account the impact of anxiety. However, their findings suggest substantial complexity in the relationship between amygdala function, CU traits and conduct problems.

To sum up, there are several limits of previous research relating CU traits, anxiety symptoms, ODD symptoms and attentional orientation toward emotional faces in children, which we aim to address in this article. Firstly, extremely few studies have taken into consideration the moderating role of anxiety in the relationship between CU traits and the processing of emotional faces. Secondly, to our knowledge, no empirical research exists addressing the question of how children with various levels of CU traits, anxiety and ODD symptoms process positive emotional faces, such as happy expressions. This inquiry is also important, since research suggests that adolescents with disruptive behaviors exhibit increased reward sensitivity (Byrd et al., 2014), and pictures of happy faces have been shown to activate reward-related brain networks (Morris et al., 1996; Phillips et al., 1998; Whalen et al., 1998). Therefore, we believe it is important to analyze in early childhood, before any clinical behavior problem is present, whether attentional orientation toward happy facial expressions is modulated by CU traits, anxiety and ODD-related problems. Finally, majority of previous studies have focused on late childhood or adolescence and on samples with conduct disorder or severe antisocial behavior. Yet, studies need to address early development and to incorporate dimensional approaches, that are not confounded by the severity of antisocial behavior or conduct disorder, in order to uncover specific ways in which CU traits, anxiety, ODD-related problems and their interactions influence attentional orientation toward emotional faces.

### Temperamental risk factors for disruptive behavior problems

Temperament traits are constitutionally-based individual differences in emotional reactivity (speed and intensity of surgency and negative affectivity) and self-regulation of emotion, which includes strategies that modulate reactivity, such as attentional control and the inhibition of dominant responses (Rothbart et al., 2006). Frick and Morris (2004) proposed that two temperamental profiles are related to childhood risk for conduct problems, through very different developmental processes. One temperamental pattern is characterized by higher reactivity, specifically higher anger, frustration and hostility in response to real or perceived provocations or novel events. This temperamental profile can act aggressively, in an emotionally dysregulated manner, within the context of these strong emotions, without thinking to the potential consequences of these acts, given the hypervigilant style of responding to emotional stimuli. Therefore, for this temperamental profile, problems in regulating high negative emotional reactivity and a hypervigilent style toward emotional stimuli increase the propensity for serious conduct problems. On the other hand, a second temperamental profile characterized by low fear (i.e., a consistent approach tendency to novel and potentially dangerous stimuli) and blunted arousal to others' distress and to punishment cues has also been linked to serious conduct problems. One of the roots that can link these two temperamental profiles to disruptive problems is the one recently proposed by Kimonis et al. (2012), who suggest that both higher and lower levels of emotional reactivity can impair the development of conscience and related complex social emotions of guilt and empathy, further increasing the risk for CU behaviors. Whereas fearless temperament can impair conscience development through insufficient engagement with important socialization cues (i.e., reduced face preference during early development; see Bedford et al., 2015), high emotional reactivity/dysregulation might make children overwhelmed in negatively charged situations, thus more prone to miss such cues in those particular contexts where they tend to be elicited (e.g., parental anger, peer distress; see Hoffman, 1982; Young et al., 1999; Frick and Morris, 2004). In short, these two different temperamental profiles would be related to CU behaviors and subsequent disruptive behavior via different emotional mechanisms.

Although Frick and Morris's (2004) model of temperamental risk factors for childhood disruptive problems is prominent, previous studies, with few exceptions (for these exceptions, see Gartstein et al., 2012; Martel et al., 2012; Wall et al., 2016; Ezpeleta et al., 2017a), did not separate temperamental reactivity from self-regulation. Also, they have largely relied on broad, undifferentiated higher-order constructs, such as negative affectivity, or on a limited number of specific temperament traits, with the majority focusing on fearlessness only. For example, recent longitudinal data (Waller et al., 2016, 2017) have linked low fear, as a precursor, to CU traits in early development. In this respect, Waller et al. (2016), using an adoption sample as well as longitudinal measures (fearlessness was measured at 18 months, CU traits and ODD at 27 months), demonstrated that biological mother's fearlessness predicted CU traits via earlier child fearlessness. Moreover, adoptive mother's positive parenting moderated the fearlessness to CU pathway. The few studies that employed more fine-grained measures of temperament, including also regulatory aspects, along with reactivity (e.g., Gartstein et al., 2012; Ezpeleta et al., 2017a), showed that children with CU traits plus ODD symptoms have deficits in self-regulation, specifically in attentional control, while those with CU traits, anxiety and ODD have significantly higher levels of negative affectivity (see Ezpeleta et al., 2017b). Moreover, Gartstein et al. (2012), in a longitudinal study that covered early childhood (from infancy till preschool period), found that higher levels of both surgency and negative emotionality predicted preschoolers' higher levels of externalizing problems, while higher levels of effortful control were linked to lower levels of externalizing difficulties. In addition, trait-by-trait moderation occurred, such that negative emotionality was most closely related to behavior problems when effortful control was low. Therefore, based on Rothbart's (2007) model of temperament that takes in consideration both the reactive and the self-regulatory dimensions of temperament, in this study we aimed to analyze, beside fear, the other fine-grained dimensions of negative affectivity (sadness, anger, discomfort), together with dimensions of surgency (activity level, approach, high intensity pleasure, impulsivity) and effortful control (attentional focusing, attentional shifting, inhibitory control), as well as with the higher order temperamental factors (negative affectivity, surgency, and effortful control), in relation to ODD-related problems, in a sample of preschool-aged children.

### Current study

The first aim of the present study was to investigate the independent contributions and the interaction effects of ODD-related problems, CU traits and anxiety on attentional orienting to emotional faces, in a community sample of preschoolers, by using a facial affect Dot-probe paradigm (MacLeod et al., 1986). This task is a widely used and effective approach to measure individual differences in attention to affective stimuli; for the present study we used it with angry, fearful, happy and neutral facial expressions. The second aim was to analyze whether both fine-grained dimensions (fear, anger, discomfort, sadness, activity level, approach, high intensity pleasure, impulsivity, attentional shifting, attentional focusing, inhibitory control) and higher order temperamental factors (negative affectivity, surgency, and effortful control) represent unique correlates of CU traits and ODD-related problems, during this time of development. In addition to independent contributions of temperament dimensions, potential moderator effects between negative affectivity and effortful control on ODD problems were also tested.

Based on previous findings on attention to emotional stimuli in children with disruptive behaviors (e.g., Kimonis et al., 2012; Hodsoll et al., 2014), we hypothesized that higher levels of CU traits would be associated with reduced attention toward fearful and angry faces, while higher levels of ODD-related problems would be associated with greater attention toward both negative and positive (happy) emotional faces. On the other hand, for the moderator effects, as seen in CU × anxiety; CU × ODD, CU × anxiety × ODD, we anticipated that high levels of CU traits and high levels of anxiety would be linked to greater attention toward angry and fearful faces, while high levels of CU traits and high levels of ODD-related problems would be associated with less orientation toward these negative emotional faces. Finally, regarding the three way interaction (CU × anxiety × ODD) we expected the effect of attentional orientation toward negative emotional faces to be most pronounced for children with high levels of CU traits, anxiety and ODD-related problems. Exploratory analyses evaluated whether CU traits, anxiety, ODD-related problems and their interactions have an impact on processing happy faces. For temperament, we predicted that high negative affectivity, high surgency and low effortful control would be associated with higher ODD-related problems. Also exploratory analyses evaluated whether these higher order temperamental factors and their subcomponents would be associated with CU traits. For the potential moderator effects between negative affectivity and effortful control on ODD problems, we predicted that high levels of negative emotionality and low levels of effortful control would be linked to ODD-related problems.

## Methods

### Participants

The sample consisted of 51 Romanian preschool-aged children (23 boys), in the age-range 53–69 months (Mean age = 63.03, *SD* = 4.69). In terms of parental education level, 26.9% of the mothers had a college degree and 21.2% had a high school degree, while 32.7% of fathers had graduated at least high school. Moreover, regarding employment status 62.7% of the mothers were employed while for the fathers the employment rate was 82.6%. Marital status data revealed that 88.5% of the parents in the sample were married.

### Measures

#### Anxiety symptoms

Child anxiety symptoms were assessed with the Spence Preschool Anxiety Scale (SCAS—Spence et al., 2001). The scale is used both with clinical and research purposes as a measure that helps identify anxiety symptoms in children. For the aim of this study we employed the Romanian parent-report version of the Spence Preschool Anxiety Scale (Benga et al., 2010) which has been translated from English to Romanian in accordance to the guidelines of the International Test Commission (van de Vijver and Hambleton, 1996). This scale consists of 28 items, coded on a five point scale from 0 (not at all true) to 4 (very often true). The SCAS items cover six sub-scales each tapping into a specific aspect of child anxiety, namely Generalized Anxiety Disorder (e.g., “Has trouble sleeping due to worrying.”); Social Anxiety (e.g., “Is afraid of meeting or talking to unfamiliar people.”); Separation Anxiety Disorder (e.g., “Has nightmares about being away from you.”); Obsessive-Compulsive Disorder (e.g., “Washes his/her hands over and over many times each day.”) and Physical Injury Fears (e.g., “Is scared of thunderstorms”). In addition, there are 1 open-ended (non-scored) item and 5 non-scored post-traumatic stress disorder items. In this study, by summing scores for all items, we computed and used the total score of the scale, since we were interested in indexing anxiety symptoms. As for psychometric properties, in the current sample Cronbach's alpha was 0.86.

#### Callous unemotional traits

For the assessment of callous unemotional traits we used the preschool version of the Inventory of Callous Unemotional Traits (ICU, Frick, 2004). In this study we used the Romanian version for teacher report which was adapted for use with Romanian teachers. The teacher version of the inventory consists of 24 items coded on a 4-point Likert scale (where 0 = not at all true and 3 = definitely true). Furthermore, the items are divided into three factors: Uncaring (e.g., “Tries not to hurt others' feelings”-reversed), Callousness (e.g., “The feelings of others are unimportant to him/her.”) and Unemotional (e.g., “Does not show emotions.”). For the present study, we used the total score which proved to have a Cronbach's α of 0.90.

#### Oppositional defiant related problems

For the measurement of ODD-related problems we used the Child Behavior Checklist (CBCL) 1½-5 years. The CBCL (Achenbach and Rescorla, 2000) is an instrument that assesses a variety of childhood emotional and behavioral problems. The Romanian version of this instrument was translated and validated on Romanian population (see Ivanova et al., 2010). In this study we used both the parent and the teacher versions and we were interested in the CBCL scoring profile drawn from DSM-referenced scales for ODD, which contains six items (Defiant, Disobedient, Angry Moods, Stubborn, Temper Tantrum, and Uncooperative). The Cronbach's α for ODD scale in the current sample was 0.82 for the parent version and 0.81 for the teacher version.

#### Temperament

In order to evaluate child temperament, we employed the Children's Behavior Questionnaire (CBQ). The CBQ (Rothbart et al., 1994, 2001; see Benga, 2004—for the Romanian version) is an evaluation of the child's temperament, responded to by the parent. This questionnaire was developed for children between the ages of 3 and 7 and consists of 195 items which can be answered on a scale from 1 to 7 (where 1 = very untrue and 7 = very true). There is also an additional option for those items that do not apply, specifically “not applicable,” resulting in score omission. All items cover three higher order temperamental dimensions: negative affectivity, surgency/extraversion and effortful control. In this study we used both these higher-order dimensions but also their fine-grained components, such as: fear (e.g., “Is not afraid of large dogs and/or other animals.”), anger/frustration (e.g., “Has temper tantrums when he/she doesn't get what he/she wants.”), discomfort (e.g., “Is not very bothered by pain.”) and sadness (e.g., “Cries sadly when a favorite toy gets lost or broken.”) for negative affectivity; activity level (e.g., “Seems always in a hurry to get from one place to another”), approach (e.g., “Gets so worked up before an exciting event that he/she has trouble sitting still.”), high intensity pleasure (e.g., “Likes going down high slides or other adventurous activities.”) and impulsivity (e.g., “Usually rushes into an activity without thinking about it.”) for surgency/ extraversion; attentional focusing (e.g., “When picking up toys or other jobs, usually keeps at the task until it's done.”), attentional shifting (e.g., “Needs to complete one activity before being asked to start on another one.”) and inhibitory control (e.g., “Can lower his/her voice when asked to do so.”) for effortful control. The score for each subscale is calculated by using the mean of items belonging to it. Adequate internal consistency indices were reported for the original CBQ scales and factors (Rothbart et al., 2001). In a Romanian validation study on 676 children (Benga, 2004), the scales included in CBQ factors had Cronbach's α values ranging between 0.56 and 0.86. For the present study, Cronbach's α were 0.72 for negative affectivity; 0.79 for surgency and 0.75 for effortful control.

#### Attentional orienting to emotional faces

As a measuring paradigm for attentional orienting to emotional faces, we used the Dot-probe task adapted from Bradley et al. (1998). Each trial of this task began with a 500 ms fixation, followed by a face pair displayed horizontally, side by side, showing human facial expressions for 500 ms. The faces were followed by the probe (a start), which replaced one of the pictures and disappeared when the participants pressed one of two keys, which were assigned to indicate the position of the probe on the screen. Children indicated as quickly and accurately as possible whether the probe appeared on the left or right side of the screen via button press (key A when the probe replaced the picture on the left side of the screen and key L when the probe replaced the picture on the right side of the screen on a QWERTY keyboard). In addition, in order to decrease the working memory load, we labeled with stickers the corresponding keys. For each child, the program presented the picture pairs in random order. In the end of each trial, a blank white screen signaled pause for 500 ms. The training stage had 6 trials within which we presented neutral stimuli from the International Affective Pictures System (Lang et al., 2008). The images used during the experimental trials were selected from the NimStim (Tottenham et al., 2009; http://www.macbrain.org/resources.htm). Therefore, from the NimStim dataset we selected face pairs of 10 actors (five female) that were displayed across 140 trials, divided into two experimental blocks. These 140 trials were split into four conditions: Happy-Neutral (40 trials), Angry-Neutral (40 trials), Fearful-Neutral (40 trials) and Neutral-Neutral (20 trials). A pair of pictures had the size of 800 × 600 pixels and each of the faces inside the pairs had 290 × 415. Images were restricted to Caucasian persons. The race constraint is due to the fact that Romanian children are mostly familiarized with these particular features. Previous investigations (Susa et al., 2014) showed that children accurately identify the emotional meaning of these facial expressions, and rate their emotional intensity, performing at adult levels. Based on the probe position, we had congruent trials where the probe appeared on the same location as the emotional face (angry, fearful, or happy), and incongruent trials where the probe appeared on the same location as the neutral face. For the neutral-neutral trials, the probe could appear in either location.

### Procedure

Children were tested with the Dot-probe task only after parents returned their written informed consent. Children individually completed the Dot-probe task in a spare resource room at the kindergarten. At the end, each child received positive feedback and a small reward. The primary caregivers (for this sample the mothers) as well as the teachers were given all the questionnaires to fill in at home. For the Dot-probe reaction time data preparation, trials with incorrect responses were excluded from the reaction time analysis. Outliers were identified as reaction times (RTs) less than 200 ms and more than 3 SD above each participant's own mean reaction time, within each experimental condition, and thus removed. This screening procedure for outliers was based on previous studies conducted with children (Waters et al., 2012). Then, based on a novel, dynamic computational methodology, proposed by Zvielli et al. (2015), we computed the Trial Level Parameters (TL-BS) to estimate attentional orientation patterns toward and/or away from emotional facial expressions from trial to trial in the Dot Probe task. TL-BS Parameters yields a series of repeated estimations of attentional orientation, toward and/or away from the faces, from trial to trial over time, per individual—rather than a single aggregated static mean estimate of attentional orientation (Zvielli et al., 2015). This computation procedure is in accordance with recent findings, demonstrating that the attentional orientation pattern toward and/or away from emotional facial expressions is more dynamic, rather than being a stable individual variable as it was traditionally conceptualized (Zvielli et al., 2015). Having chosen this approach, we computed the TL-BS scores that allow the quantification of three new parameters of attentional orientation in relation to each emotional face (angry, fearful, and happy). These parameters reflect the individual differences pertaining to the expression of trial-level attentional orientation. During the computation of TL-BS, for each individual, we first matched congruent (where the probe appeared on the same location as the emotional face) and incongruent (where the probe appeared on the same location as the neutral face) trial response times with the corresponding neutral response time (RT). Then, we subtracted the neutral RT from the congruent RT and the incongruent RT from the neutral RT. The differences were afterwards used to calculate the parameters (see Table 1 for descriptive statistics). The first parameter is Mean TL-BS and is a bi-dimensional parameter, calculated twofold for each of the congruent and incongruent set of trials, in the case of each participant; Mean TL-BS positive (Mean TL-BS Toward) indicates individual differences in the degree to which the attention is oriented toward the emotional face or to which the mean TL-BS > 0 ms, whereas Mean TL-BS negative (Mean TL-BS Away) reflects individual differences in the degree to which the attention is oriented away from the emotional face or the degree to which TL-BS < 0 ms. The second parameter is Peak TL-BS, which is also bi-dimensional and calculated twofold; Peak TL-BS positive (Peak Toward) shows the individual differences in the maximum phasic expression of the trial-level orientation toward the emotional face, while Peak TL-BS negative (Peak Away) indicates the individual differences in the maximum phasic expression of the trial-level attention away from the emotional face. Lastly, Variability in TL-BS is calculated by using the standard deviation formula for each set of differences. This parameter points to the degree of stability or temporal variability in the manifestation of the attentional orientation over time toward and/or away from the relevant stimuli (Zvielli et al., 2015).

**Table 1:** Descriptive statistics for TL-BS parameters for each emotional face, CU traits, anxiety and ODD-related problems.

**Variable**	**Mean**	***SD***
Angry mean away	−503.78	1, 308.87
Angry mean toward	470.76	1, 065.09
Angry peak away	−1, 372.98	2, 321.73
Angry peak toward	1, 529.20	2, 832.77
Angry variability	680.67	1, 405.15
Fearful mean away	−412.91	662.87
Fearful mean toward	1, 130.42	5, 760.62
Fearful peak away	−1, 501.10	2, 775.46
Fearful peak toward	2, 610.63	8, 902.11
Fearful variability	1, 238.36	4, 956.55
Happy mean away	−991.91	4, 340.92
Happy mean toward	408.73	700.61
Happy peak away	−2, 500.53	8, 806.67
Happy peak toward	1, 530.69	2, 920.52
Happy variability	1, 039.21	3, 578.84
CU traits	32.25	9.17
Anxiety symptoms	23.23	15.13
ODD teacher	1.38	1.99
ODD parent	3.35	2.78

## Results

### Regression analysis for independent contributions and interaction effects of ODD-related problems, CU traits and anxiety on attentional orienting to emotional faces

**Figure 1:**
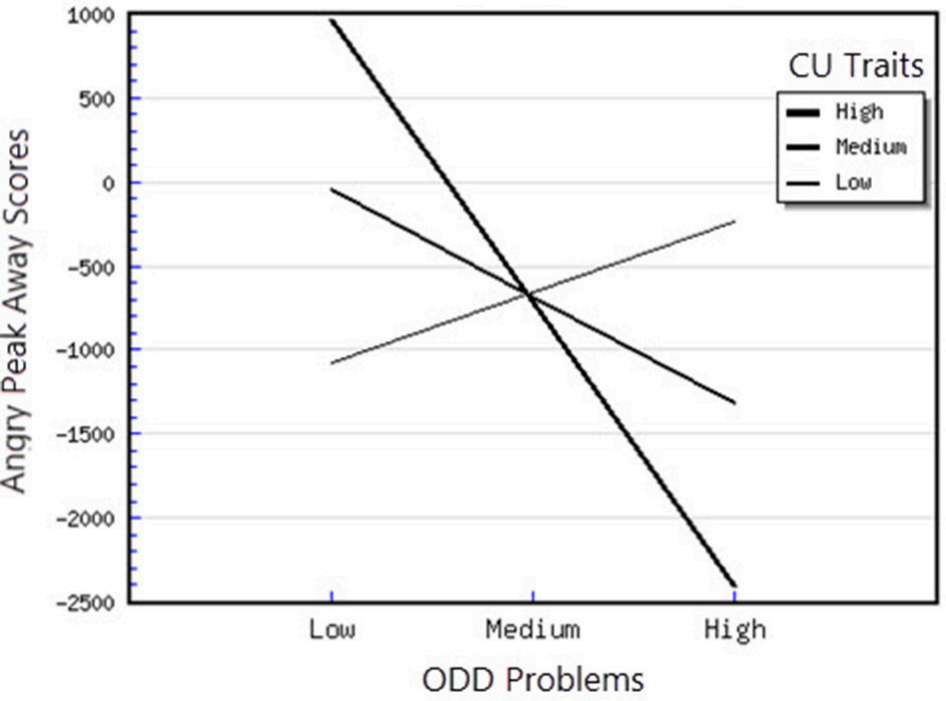
Interaction between ODD problems and CU traits in predicting angry peak away scores.

**Figure 2:**
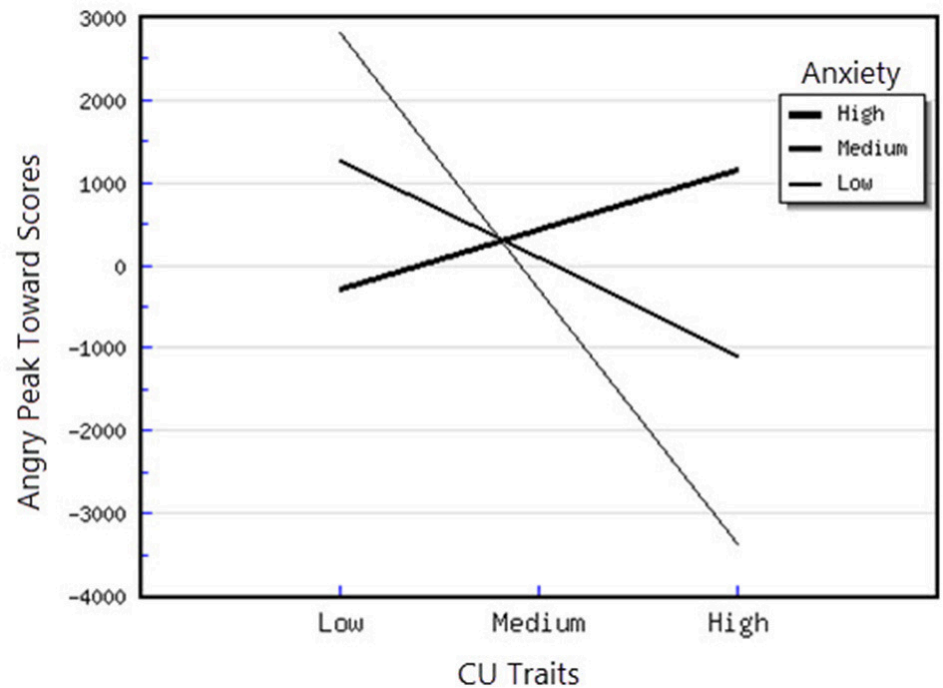
Interaction between CU traits and anxiety in predicting angry peak toward scores.

**Figure 3:**
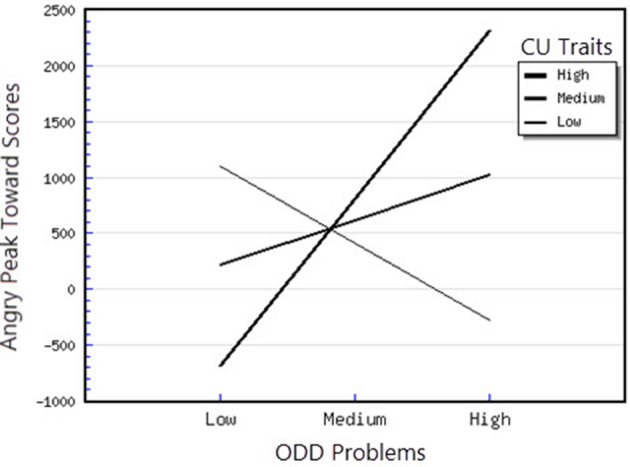
Interaction between ODD problems and CU traits in predicting angry peak toward scores.

**Figure 4:**
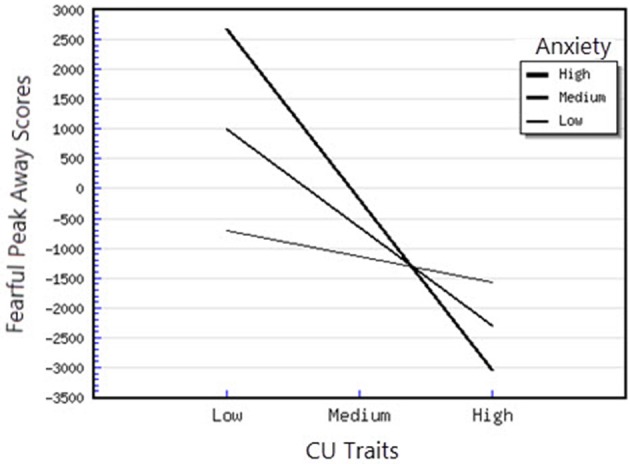
Interaction between CU traits and anxiety in predicting fearful peak away scores.

**Figure 5:**
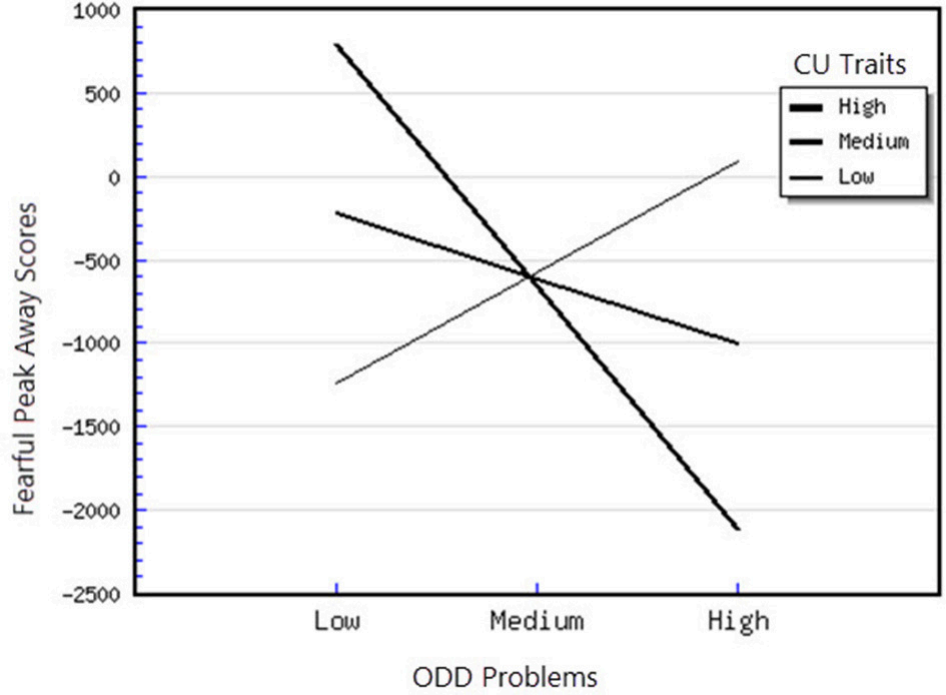
Interaction between ODD problems and CU traits in predicting fearful peak away scores.

**Figure 6:**
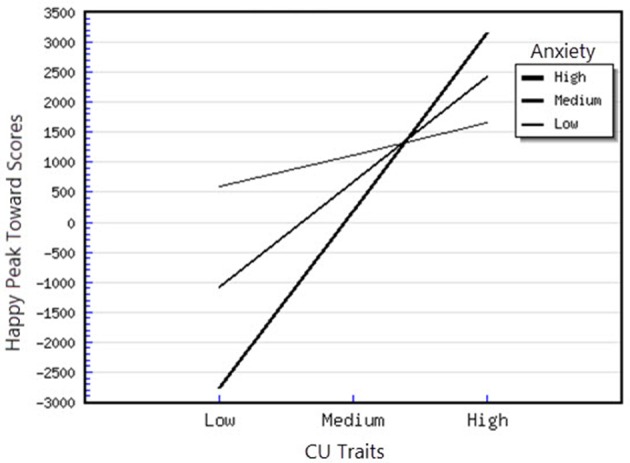
Interaction between CU traits and anxiety in predicting happy peak toward scores.

**Figure 7:**
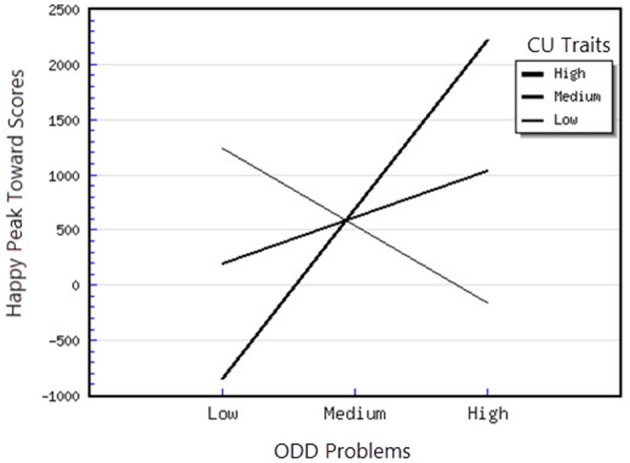
Interaction between ODD problems and CU traits in predicting happy peak toward scores.

Hierarchical multiple regressions were run to assess unique and interactive relations between ODD-related problems, CU traits, anxiety and attentional processing of emotional faces. However, before conducting these regressions, we analyzed the bivariate correlations between these variables of interest. As it can be seen in Table 2, CU traits and ODD-related problems rated by teachers were significantly and negatively associated with Peak Away scores and positively associated with Peak Toward scores in the case of angry faces. In the case of fearful faces, CU traits and ODD-related problems rated by teachers were also both significantly and negatively related to Peak Away scores. For happy faces, both CU traits and ODD teacher rates were significantly and positively associated only with Peak Toward scores. Therefore, given that only these four parameters were significantly associated with CU traits and ODD problems (teacher rate), we further conducted four separate multiple hierarchical regression analyses, one for each of these parameters, in order to examine the contributions of CU traits, anxiety, ODD-related problems and their interactions on attentional processing of emotional faces as indexed by these parameters. In each regression, CU traits, anxiety and ODD-related problems as rated by teachers were entered in the first step, while the two-way interactions in the form of multiplicative products of these centered variables (CU traits × Anxiety; CU traits × ODD; Anxiety × ODD) were entered in the second step. Finally, in the last step, the three-way interaction between CU traits × Anxiety × ODD was entered (see Table 3 for the results of the regression analysis). For the Angry Peak Away parameter, the model in which all predictors were included explained 57% of the variance in individual differences in phasic bursts that reflect amplitudes of attentional avoidance of angry faces. This result indicates that the entire model has a large effect (*f* ^2^ = 1.32) on the outcome variable. As expected, the interaction between CU traits and ODD-related problems was significant, indicating the presence of a moderation effect. To interpret this significant interaction, the regression coefficients of ODD-related problems upon the Angry Peak Away scores were inspected at 1 SD above and below the mean of the CU traits (the moderator factor), as recommended by Aiken et al. (1991). The slope was significantly different from zero only at high levels of CU traits *t*_(48)_ = −2.52, *p* < 0.05. Therefore, for children with high levels of CU traits, higher levels of ODD-related problems were related to greater attentional avoidance of angry faces (see Figure 1). Moreover, for the Angry Peak Toward parameter, the model in which all predictors were included explained 53% of the variance in individual differences in attentional phasic bursts (amplitudes) toward angry faces. This result indicates that the entire model has a large effect (*f* ^2^ = 1.12) on the outcome variable. As expected, the interaction between CU traits and anxiety, as well as between CU traits and ODD-related problems were significant. Examination of these interactions in predicting the Peak Toward scores for angry faces revealed that, for the moderator effect of anxiety, the slope was significantly different from 0 at low levels of anxiety *t*_(48)_ = −3.14, *p* < 0.05, showing that for children with lower levels of anxiety, CU traits were associated with less attention orientation toward angry faces (see Figure 2). Moreover, for the interaction between CU traits and ODD-related problems, the slopes were significantly different from 0 at high [*t*_(49)_ = 2.21, *p* < 0.05] levels of CU traits. Thus, at high levels of CU traits, ODD-related problems were significantly and positively associated with attentional orientation toward angry faces (see Figure 3). In the case of Fearful Peak Away parameter, the final model in which all predictors were included explained 56% of the variance in individual differences in phasic bursts (amplitudes) of attentional avoidance of fearful faces. This result indicates that the entire model has a large effect (*f* ^2^ = 1.27) on the outcome variable. CU traits were positively and significantly associated with the Fearful Peak Away parameter (*B* = 108.50, *p* < 0.05), however this association was further moderated by anxiety, as the interaction between CU traits and Anxiety was significant. Examination of this interaction demonstrated that the slope was significantly different from 0 at both high levels of anxiety, *t*_(48)_ = −4.21, *p* < 0.01 and medium levels of anxiety *t*_(48)_ = −3.48, *p* < 0.01, showing that, in this case, higher CU traits were significantly related to greater attentional avoidance of fearful faces (see Figure 4). Moreover, the interaction between CU traits and ODD-related problems was also significant. For this interaction, we found that that the slope was significantly different from 0 at high levels of CU traits, *t*_(48)_ = −2.30, *p* < 0.05, showing that for children with high levels of CU traits, higher ODD-related problems were significantly associated with higher avoidance of fearful faces (see Figure 5). Finally, for the Happy Peak Toward parameter, we found that the model in which all predictors were included explained 52% of the variance in individual differences in attentional phasic bursts (amplitudes) of orientation toward happy faces. Therefore, this model has a large effect (*f* ^2^ = 1.08) on the outcome variable. Also, for this parameter, the interaction effects between CU traits and Anxiety, as well as between CU traits and ODD-related problems were significant. As we did for the other interactions, we examined the direction of these effects by plotting the regression of the Happy Peak Toward scores on 1 *SD* above and below the mean of the CU traits and Anxiety (see Figures 6, 7). For the interaction between CU traits and Anxiety, the slope was significantly different from 0 at high levels of anxiety *t*_(48)_ = 4.03, *p* < 0.01 and medium levels of anxiety *t*_(48)_ = 3.46, *p* < 0.01 suggesting that for children with high and medium levels of anxiety, higher CU is associated with greater attentional orientation toward happy faces. Moreover, for the CU × ODD interaction, the simple slope analysis revealed that the slope was significantly different from 0 at high levels of CU traits, *t*_(48)_ = 2.21, *p* < 0.05, showing that for children with high CU traits, higher ODD-related problems were significantly and positively associated with attentional orientation toward happy faces.

**Table 2:** Bivariate correlations between TL-BS parameters for each emotional face, CU traits, anxiety and ODD-related problems.

	**Angry mean away**	**Angry mean toward**	**Angry peak away**	**Angry peak toward**	**Angry variability**	**Fearful mean away**	**Fearful mean toward**	**Fearful peak away**	**Fearful peak toward**	**Fearful variability**	**Happy mean away**	**Happy mean toward**	**Happy peak away**	**Happy peak toward**	**Happy variability**
CU traits	−0.07	0.13	−0.31^*^	0.41^**^	0.25	−0.26	−0.05	−0.47^**^	0.06	0.02	0.02	0.11	−0.04	0.44^**^	0.05
ODD teacher	−0.12	0.13	−0.40^**^	0.30^*^	0.26	−0.25	−0.07	−0.35^*^	0.02	0.00	0.06	0.05	0.03	0.34^*^	−0.01
ODD parent	0.08	−0.04	0.04	0.16	0.00	−0.03	−0.07	−0.16	0.00	−0.04	0.05	−0.05	0.02	0.14	−0.02
Anxiety	0.13	−0.10	0.04	0.17	−0.02	−0.04	−0.13	−0.22	−0.05	−0.10	0.11	−0.10	0.06	0.20	−0.07

* p ≤ 0.05;

** *p ≤ 0.01*.

**Table 3:** Hierarchical regression analysis for variables predicting TL-BS parameters.

**Variable**	**Angry peak away**			**Angry peak toward**			**Fearful peak away**			**Happy peak toward**		
	**B**	**SE B**	**β**	**B**	**SE B**	**β**	**B**	**SE B**	**β**	**B**	**SE B**	**β**
ODD problems	386.77	201.23	0.33	−138.97	244.07	−0.09	174.44	230.22	0.13	−195.97	245.64	−0.14
Anxiety	−8.91	21.02	−0.06	−19.26	25.50	−0.10	25.58	24.06	0.14	−24.49	25.67	−0.13
CU traits	27.30	45.59	0.11	−100.88	55.30	−0.32	108.50^*^	52.16	0.36	−106.37	55.66	−0.33
CU traits × anxiety	−1.31	1.84	−0.08	8.98^**^	2.45	0.47	−8.37^**^	2.26	−0.44	8.38^**^	2.47	0.42
CU traits × ODD	−57.70^**^	9.91	−0.90	51.28^**^	13.16	0.65	−51.40^**^	12.15	−0.66	54.33^**^	13.29	0.67
Anxiety × ODD	−11.76	11.22	−0.11	−8.93	14.90	−0.07	7.65	13.77	0.06	−9.66	15.05	−0.07
CU traits × anxiety × ODD	−0.01	1.44	0.00	3.11	1.86	0.25	−2.42	1.73	−0.20	2.43	1.90	0.19
*R*_2_	0.57			0.53			0.56			0.52		

* p ≤ 0.05;

** *p ≤ 0.01*.

### Correlations and regression analysis for the association between temperamental factors, CU traits and ODD problems

In order to analyze whether both fine-grained dimensions and higher order temperamental factors represent unique correlates of CU traits and ODD-related problems, Pearson correlations were run (see Table 4). These analyses revealed that attention focusing, as a fine-grained dimension of self-regulative effortful control, was significantly and negatively associated with CU traits. Moreover, the higher order temperamental factor of effortful control was also significantly and negatively associated with CU traits. ODD-related problems, as rated by teachers, were significantly and negatively associated with attention shifting. Also, the higher order temperamental factor of effortful control was significantly and negatively associated with ODD problems as rated by teachers. For ODD-related problems rated by parents, we found significant and positive associations with the following fine-grained dimensions of temperamental reactivity: high intensity pleasure, impulsivity, sadness and frustration. In addition, higher order temperamental factors of negative affectivity and surgency were significantly and positively related to parental ratings of ODD-related problems. For fine-grained dimensions of temperamental self-regulation we found that parental ratings of ODD-related problems were significantly and negatively associated with attention shifting and inhibitory control. Furthermore, the higher order temperamental factor of effortful control was also significantly and negatively associated with ODD parent rate. In order to test the potential moderator effect between negative affectivity and effortful control on ODD-related problems, we conducted two separate multiple hierarchical regression analyses, one for the parental and the other for the teacher rate of ODD-related problems. For both regressions, there was no evidence of significant interactions between negative affectivity and effortful control (*B* = 0.73, *p* = 0.73 for the parent rate; *B* = −0.95, *p* = 11, for teacher rate) in predicting ODD-related problems.

**Table 4:** Bivariate correlations between temperamental factors, ODD-related problems and CU traits.

	**Activity level**	**High intensity pleasure**	**Impulsivity**	**Surgency**	**Fear**	**Sadness**	**Frustration**	**Discomfort**	**Negative affectivity**	**Attention focusing**	**Attention shifting**	**Inhibitory control**	**Effortful control**	**ODD parent**	**ODD teacher**	**CU traits**
Activity level																
High intensity pleasure	0.47^**^															
Impulsivity	0.57^**^	0.53^**^														
Surgency	0.83^**^	0.81^**^	0.84^**^													
Fear	−0.09	−0.15	−0.04	−0.11												
Sadness	0.25	0.43^**^	0.33^*^	0.41^**^	0.25											
Frustration	0.36^**^	0.50^**^	0.58^**^	0.58^**^	0.20	0.66^**^										
Discomfort	−0.08	0.03	−0.14	−0.08	0.51^**^	0.45^**^	0.33^*^									
Negative affectivity	0.13	0.25	0.23	0.25	0.69^**^	0.77^**^	0.72^**^	0.79^**^								
Attention focusing	−0.16	−0.13	−0.40^**^	−0.27^*^	−0.01	−0.10	−0.38^**^	−0.02	−0.17							
Attention shifting	−0.33^*^	−0.38^**^	−0.53^**^	−0.50^**^	−0.17	−0.56^**^	−0.60^**^	−0.33^*^	−0.54^**^	0.33^*^						
Inhibitory control	−0.42^**^	−0.24	−0.51^**^	−0.47^**^	−0.09	−0.32^*^	−0.50^**^	−0.14	−0.35^*^	0.64^**^	0.54^**^					
Effortful control	−0.37^**^	−0.28^*^	−0.60^**^	−0.50^**^	−0.10	−0.40^**^	−0.61^**^	−0.19	−0.43^**^	0.81^**^	0.76^**^	0.88^**^				
ODD parent	0.16	0.37^**^	0.44^**^	0.39^**^	0.05	0.40^**^	0.43^**^	0.19	0.35^*^	−0.24	−0.48^**^	−0.41^**^	−0.45^**^			
ODD teacher	0.16	0.06	0.17	0.16	−0.14	0.23	0.23	0.14	0.15	−0.26	−0.27^*^	−0.16	−0.30^*^	0.15		
CU traits	0.03	−0.03	0.14	0.05	−0.07	0.14	0.09	0.24	0.13	−0.28^*^	−0.20	−0.17	−0.28^*^	0.23	0.61^**^	

* p ≤ 0.05;

** *p ≤ 0.01*.

## Discussion

The first objective of the present investigation was to analyze, in a sample of preschool children, the independent contributions and the interaction effects of CU traits, anxiety and ODD-related problems on attentional orienting to emotional faces. In this respect, we report a number of key findings.

### Attentional orienting to emotional faces as a function of ODD-related problems, CU traits and anxiety

First, attentional orientation patterns, as indexed through TL-BS parameters, revealed that the direction of peaks (phasic expression of the trial-level orientation, which can be toward or away from emotional faces—Peak Toward, respectively Peak Away) varied, based on the interactions between individual differences in CU traits and ODD-related problems. Specifically, we found that relationships between ODD-related problems, on the one side, and Angry Peak Away, Angry Peak Toward, Fearful Peak Away and Happy Peak Toward, on the other side, were moderated by levels of CU traits. Thus, for the Angry Peak Away parameter, at higher levels of CU traits, higher levels of ODD-related problems were significantly associated with greater attentional avoidance of angry faces. Additionally, for Angry Peak Toward parameter, at higher levels of CU traits, higher levels of ODD-related problems were significantly associated with greater attention toward angry faces, while for Fearful Peak Away, with higher avoidance of fearful faces. For Happy Peak Toward, we found that, at higher levels of CU traits, ODD-related problems were significantly related to greater attentional orientation toward positive faces. In the case of negative emotional stimuli, these results partially support our hypothesis regarding the interaction effects of CU traits and ODD on attentional allocation. Specifically, our prediction was that children with combined CU traits and oppositional related problems would orient attention less toward negative emotional faces, especially fearful ones. Our results demonstrated that, for the Fearful Peak Away parameter, children with combined high levels of CU traits and ODD-related problems had greater avoidance of fearful faces. This result is consonant with previous data, showing that children with CU traits and ODD-related problems are less sensitive to emotions that reflect others' distress, such as fear and sadness (Blair et al., 2001; Kimonis et al., 2012, 2016). Less sensitivity to others' distress as indexed by attentional avoidance of fearful faces has been suggested to facilitate a lack of inhibition of aggressive behaviors. This interpretation is based on studies with typically developing individuals, showing that they tend to interpret fear and sadness in others as aversive; thus, when an aggressive act is carried out and an expression of fear or sadness observed, this act is perceived to be aversive and it is inhibited, via classical conditioning (Blair et al., 2001; Ezpeleta et al., 2017b). However, our findings on angry faces have revealed contradictory results as to whether CU traits and ODD-related problems are associated (or not) with attentional avoidance. Particularly, we showed that both attentional allocation toward and away from these facial stimuli were predicted by high CU traits and high ODD-related problems. Therefore, these data point that, in relation to angry faces, children with combined CU traits and ODD-related problems can show either attentional facilitation or avoidance of these stimuli. The scarce literature on attention to angry faces in children with non-clinical levels of ODD problems and CU traits makes it difficult to link our results with previous data. However, these previous results also report divergent findings, that range from attentional avoidance (see Hodsoll et al., 2014, who found that boys aged 8–16 with clinical levels of conduct problems and high levels of CU showed reduced attentional capture by angry faces) to attentional orientation toward angry faces (see Ezpeleta et al., 2017b, who showed that children with high but non-clinical levels of CU traits and ODD-related problems oriented their attention to angry faces to the same degree as children with low CU traits and low ODD-related problems, during an emotional version of the Go/No-Go task). Moreover, data on emotional face recognition proved that CU traits with conduct problems have also been associated with better accuracy in identifying angry faces (Wolf and Centifanti, 2014). For happy faces, we did not formulate a specific hypothesis, since our investigation was exploratory regarding positive stimuli processing. Nevertheless, greater attention orientation toward happy faces, for children with high CU traits and high ODD-related problems, is in line with data suggesting that adolescent youth with disruptive behaviors exhibit increased reward sensitivity (Byrd et al., 2014) and that CU traits are associated with a tendency to be over-focused on reward (Frick et al., 2003; Frick and White, 2008).

Second, the direction of peaks also varied based on the interactions between individual differences in CU traits and anxiety symptoms. Specifically, we demonstrated that the relationships between CU traits, Angry Peak Toward, Fearful Peak Away and Happy Peak Toward were moderated by levels of anxiety symptoms. Thus, for the Angry Peak Toward parameter, at lower levels of anxiety, higher CU traits were significantly associated with less attentional orientation toward angry faces. Moreover, the combination of CU traits and anxiety was associated with greater avoidance of fearful faces. So, contrary to our expectation and previous developmental data (see Kimonis et al., 2012—however, compared to our study, these authors focused on male adolescents with combined anxiety and conduct disorder and used emotionally distressing pictures from IAPS, instead of faces), the presence of CU traits in combination with higher anxiety was not associated with greater orientation toward distress stimuli, such as fearful faces. In contrast, our data revealed that the presence of higher CU traits is related to attentional avoidance of fearful faces for higher levels of both anxiety and ODD-related problems. Support for this association between CU traits and processing of fearful faces comes from neurological studies of older children having CU traits, that report reduced amygdala activation while processing fearful, but not angry faces, as compared to typical child samples (Marsh and Blair, 2008). Theories of moral socialization (Fowles and Kochanska, 2000; Bedford et al., 2015) suggest that blunted reactivity to others' distress, as reflected by avoidance in processing fearful faces, can inhibit the typical development of morality and conscience. This can unfold through insufficient engagement with important socialization cues, such as others' feelings and punishment, leading to reduced learning about the outcomes of harmful behaviors, and, as a result, to higher antisocial responses. For Happy Peak Toward, we found that, at higher and medium levels of anxiety, higher CU traits were associated with greater attentional orientation toward happy faces, indicating that these children, similarly to those with CU traits and ODD-related problems, are characterized by higher reactivity to positive emotional stimuli.

### Associations between temperamental traits, CU traits and ODD-related problems

Our second objective was to analyze whether fine-grained dimensions of reactivity (fear, anger, discomfort, sadness, activity level, approach, high intensity pleasure, impulsivity) and self-regulation (attentional shifting, attentional focusing, inhibitory control), as well as the higher order temperamental factors (negative affectivity, surgency, and effortful control) represent unique correlates of CU traits and ODD-related problems. While most approaches involving temperament have focused on the higher factor of negative affectivity or on its subdimension of fear (Waller et al., 2016, 2017), while not separating temperamental reactivity from self-regulation, our analysis considered, probably for the first time in preschool population, the contributions of both fine-grained dimensions and higher order temperamental factors, for temperamental reactivity as well as for self-regulation. For the fine-grained components of effortful control—the self-regulative temperamental dimension—we found that higher attentional focusing was related to lower levels of CU traits. Thus, this temperamental factor could be playing a protective role for the expression of CU traits. Also, higher effortful control was related to lower levels of CU traits; however, attentional focusing was largely responsible for the link between effortful control and CU traits, since no other fine-grained components of effortful control were found to be associated with this variable. Our result further supports the importance of attentional abilities in promoting adaptive development (Lonigan and Phillips, 2001; Calkins and Degnan, 2006). Moreover, the protective role of temperamental effortful control was also evident in relationship to ODD-related problems. As we expected, and in line with previous developmental data (Gartstein et al., 2012), higher levels of effortful control were associated with lower ODD-related problems, as indicated by both parent and teacher ratings. At the fine-grained level of analysis, all components of effortful control (attentional shifting, attentional focusing, and inhibitory control) were related to fewer parent-reported ODD-related problems. These links could be explained based on the assumption that willful control of attention and inhibitory control may allow children to redirect their focus away from distressing stimuli, in order to dampen negative emotions and to inhibit their dominant responses (Gartstein et al., 2012). Moreover, additional components of negative affectivity (sadness, frustration) and of surgency (high intensity pleasure and impulsivity) were related to higher levels of parental ratings of ODD-related problems. In addition to independent contributions of temperament traits, potential moderator effects, as seen in negative affectivity and effortful control interactions, were tested on ODD-related problems. Our data revealed no evidence of significant interactions between negative affectivity and effortful control in predicting ODD-related problems. Few studies have examined trait-by-trait moderation on behavioral problems and their results are inconsistent. Specifically, Gartstein et al. (2012), in a longitudinal study found that that negative emotionality was most closely related to behavior problems when effortful control was low while in a recent study, conducted with clinically referred children and general population sample, Scheper et al. (2017) demonstrated no evidence for such moderation.

### Strengths and limitations

The present study contributes to the literature by including a community sample of preschoolers and incorporating dimensional measures of CU traits, anxiety symptoms and ODD-related problems. This was critical, since most studies so far have focused on older children and adolescents with conduct disorder or severe antisocial behavior, leaving the possibility that the severity of antisocial behavior may be responsible for the observed effects. Moreover, our study included measures provided by teachers for the CU traits and ODD-related problems, as compared to previous studies that predominantly used parents and children as reporters. Teachers are considered to be good observers, since the social behavior as covered by CU traits and ODD-related problems measures is considered to be more easily and reliably observed in formal educational settings (kindergartens and schools). Furthermore, to our knowledge, the present study is the first addressing the question on how children with various levels of CU traits, anxiety and ODD-related problems process both negative and positive emotional faces, by indexing attentional orientation patterns toward these stimuli through a dynamic computation procedure. This procedure permitted us to estimate attentional orientation, toward and/or away from the faces, from trial to trial over time, per individual. Finally, our data on temperament, CU traits and ODD-related problems highlight the importance of broadening the analysis of early temperamental vulnerability factors beyond fearlessness, by also considering protective factors, such as effortful control, and its subcomponents.

Limitations, however, should also be taken into account when interpreting the present results. One of the main limitations is that we used a cross-sectional correlational design, thus being unable to assess the directionality of the observed effects. Another limitation is that our sample was not large enough and this may have contributed to some of our non-significant results. Moreover, psychopathology is not very frequent in young samples from the general population, so the very age of our sample could have affected the emergence of more associations.

Despite these limitations, results of the present study have several implications. First, our data demonstrate that different patterns of attentional processing related to emotional facial expressions may characterize distinct subgroups of young children with oppositional problems, based on their CU traits and anxiety symptoms levels. This suggests that interventions should take into account the demonstrated heterogeneity and provide more personalized treatments. For example, our results suggest that for fearful faces, all children with CU traits would benefit from an intervention that train attentional allocation toward such stimuli, in order to facilitate higher sensitivity for others' distress. However, in the case of angry faces, this approach would not be effective for all children. Specifically, for children with high CU traits and ODD-related problems, that had greater attentional orientation toward angry faces, intervention approaches which focus on teaching emotion regulation techniques (that children can implement when they perceive such cues signaling anger) might be more effective. Second, our findings on temperament may be useful for early intervention and prevention efforts targeting children who fit the profile of elevated risk, in terms of their temperament, for future development of CU traits and ODD-related problems.

## Ethics statement

This study has been approved by Ethics Committee of Babes-Bolyai University. Written informed consent was obtained from all parents and/or guardians of each participating child included in the study.

## Author contributions

GS and OB contributed equally to this work (contributed to study design, data collection and processing, statistical analysis, interpretation, paper writing and review). CM contributed to data collection, processing, statistical analysis and paper writing.

### Conflict of interest statement

The authors declare that the research was conducted in the absence of any commercial or financial relationships that could be construed as a potential conflict of interest.
